# Incidental uptake of fluorodeoxyglucose in the Waldeyer’s ring and risk of oropharyngeal malignancy

**DOI:** 10.1007/s00405-021-07089-6

**Published:** 2021-09-27

**Authors:** Iulia Bujoreanu, Dorothy Gujral, Kathryn Wallitt, Zaid Awad

**Affiliations:** 1 Head and Neck Unit, Imperial College Healthcare NHS Trust, Fulham Palace Road, London, W6 8RF UK; 2 Imperial College London, South Kensington, London, SW7 2BU UK; 3 Department of Nuclear Medicine and Radiology, Imperial College Healthcare NHS Trust, Fulham Palace Road, London, W6 8RF UK

**Keywords:** Head and neck, Positron emission tomography, Oropharynx, Head and neck cancer

## Abstract

**Purpose:**

Fluorodeoxyglucose (FDG) positron emission tomography (PET) is increasingly used to diagnose and stage malignancy. The aim of this article is to investigate the significance of incidental FDG uptake in the Waldeyer’s ring and to assess its value in predicting clinically occult oropharyngeal malignancy.

**Methods:**

All FDG-PET/CT scans performed in Imperial College NHS Foundation Trust, UK between January 2012 and November 2018 were included. Patients with known or suspected oropharyngeal malignancy or lymphoma were excluded. Minimum follow-up was 12 months.

**Results:**

A total of 724 scans revealed oropharyngeal uptake of FDG. Of these, 102 were included in the study. Most patients (62.1%) were scanned as part of staging for other malignancies. Oropharyngeal FDG uptake was asymmetrical in 57.3% of the cases. Uptake was more common in the tonsils (56.3%), followed by the tongue base (31.1%) and both sites (12.6%). In 41.7% of reports, appearance was described as likely physiological; however, 52.4% of reports advised direct visualisation, clinical correlation or ENT opinion. Only 24.3% (25/102) of patients were referred and seen by ENT, 14.6% (15/102) of which had an interval PET scan and 8.7% (9/102) proceeded to tissue diagnosis. There was one oropharyngeal cancer identified and one unexpected metastasis from esophageal cancer.

**Conclusion:**

Incidental uptake on PET/CT in the oropharynx is common. However, malignancy is rare (1.9%) and, when present, is associated with high SUVmax and asymmetrical uptake. Imaging results must be correlated clinically. These patients should be seen by an ENT specialist yet most may not require further investigations.

## Introduction

Head and neck malignancy represents 3% of all newly diagnosed cancers in the UK, with approximately 11 945 cases diagnosed every year [1]. The most common histologic type is squamous cell carcinoma (SCC). Despite substantial advancements in surgical techniques and pharmacological therapies over the past decades, the prognosis of most head and neck cancers remains poor. This is partly because of advanced disease at the time of presentation in over 60% of patients. Moreover, synchronous and metachronous malignancies are common due to the phenomenon of ‘field cancerisation’ in the upper aerodigestive tract and the effect of tobacco smoking and alcohol consumption [2–4]. These findings are supported by a recent meta-analysis showing that patients with hypopharyngeal and oropharyngeal malignancies are at the highest risk of concurrent malignancies [5].

Positron emission tomography (PET) with fluorodeoxyglucose (FDG) utilises whole-body imaging to visualise the uptake of glucose and subsequent glycolysis within cells. PET imaging demonstrates metabolically active tissues and detects metabolic abnormalities prior to morphological changes [6]. Combined with structural imaging modalities such as computed tomography (CT) or magnetic resonance imaging (MRI), it provides functional and anatomical information. Standardised uptake values (SUV) have been developed to semi-quantitatively measure FDG uptake related to surrounding tissues, which considers the measured activity in a region of interest (ROI), the amount of tracer injected and patient weight.

Within the head and neck there are several areas that have physiologic increased uptake which can make interpretation of these images challenging. Furthermore, physiologic uptake can be asymmetric. Common sites include the lymphoid tissue within the Waldeyer’s ring, the extraocular muscles, the muscles of mastication and the three major salivary glands. High uptake in the vocal cords and the tongue can be associated with phonation at the time of FDG administration. Uptake in the lymphoid oropharyngeal tissue is typically increased in children and young adults. These areas of physiologic FDG uptake in the head and neck have been often documented in patients with various malignancies, such as lung cancer and haematological malignancies [7–9]. Additionally, previous chemotherapy, radiation therapy and surgery, or any other cause of active inflammation can also cause increased FDG uptake, which can appear asymmetrical [10]. The aim of this article is to investigate the significance of incidental FDG uptake in the Waldeyer’s ring and to assess its value in predicting occult oropharyngeal malignancy.

## Materials and methods

### Patients

All FDG-PET/CT scans performed and reported at Imperial College NHS Trust, UK between 1st January 2012 and 1st December 2018 were scanned for the keywords “tonsil”, “tongue” and “lingual”. The selected reports were then fully read to ascertain whether there was any uptake in the region of interest (tonsils or base of the tongue) and to collect clinical and radiological data. Collected data included gender, indication for PET scan, topography of uptake, asymmetry, SUV_max_ in the region of interest and recommendations in the official radiology report. The medical records were subsequently analysed for potential appointments with the otolaryngology service, further investigations and tissue biopsies. Data for the study were collected between the 1st November 2018 and 30th November 2019. Incidental uptake rates on FDG/PET/CT scans were calculated from January 2015 onwards as data for total numbers of scans performed in our unit was not available prior to this date. The study did not require ethics committee approval.

### Inclusion and exclusion criteria

Included patients were older than 18 years and had unexpected or incidental FDG uptake in the oropharynx during a PET/CT scan. This was either during a whole body imaging study or during a dedicated head and neck scan. Patients with physiological uptake were included. Exclusion criteria included age less than 18 years, a current or previous diagnosis of head and neck malignancy apart from thyroid cancer or haematological malignancy and lack of FDG uptake in the region of interest. Patients were also excluded if their medical records were not available for review, if they passed away before having the opportunity to investigate the incidental FDG uptake or if they did not turn up for further investigations.

### Imaging protocol and image analysis

Cross-sectional imaging was acquired using a Siemens Biograph™ TruePoint TrueV PET scanner (Munich, Germany) with true X reconstruction. Patient were asked to fast for a minimum of 6 h prior to their radiology appointment and capillary blood glucose levels were measured using a point-of-care device prior to the administration of radioisotope. FDG was administered via a peripheral intravenous line. The images were then reviewed and reported by a nuclear medicine consultant radiologist.

### Follow-up

Patients were followed-up for development of any late head and neck malignancy for a minimum time of 12 months, starting from the time of their first FDG-PET/CT with incidental oropharyngeal uptake.

### Clinical outcomes

The main measured clinical outcome was whether any of the patients with incidental oropharyngeal uptake had a concurrent head and neck malignancy. The secondary outcome looked at whether any of the included patients developed a head and neck malignancy during the follow-up period. Data were presented as descriptive statistics, using mean and standard deviation for normally distributed data, and median and range for not normally distributed data. Ratios were presented as percentages.

## Results

Incidental uptake rates varied between 0.64 and 1.42% of all PET CT scans per year with an average of 0.88% (Table 1). In a period of 82 months, a total of 724 patients showed head and neck FDG uptake. From these, 434 patients were excluded for having a suspected or previous diagnosis of head and neck malignancy excluding thyroid cancer. A further 135 patients were excluded for having or being suspected of having a haematological malignancy, 45 patients were excluded as they did not have any FDG uptake in the lingual or palatine tonsils and seven patients were excluded as there was full clinical notes were not available. One patient was not included in the study as he declined further investigations and was lost to follow-up. Finally, 102 patients were included in the analysis (Table 2).

**Table 1:** Results—rates of incidental uptake

Year	Total FDG-PET/CT scans performed	FDG-PET/CT scans with incidental oropharyngeal uptake (%)
2015–2016	2639	17 (0.64%)
2016–2017	2799	22 (0.79%)
2017–2018	3015	20 (0.66%)
2018–2019	1899	27 (1.42%)

**Table 2:** Results—demographics and basic clinical characteristics and radiographic findings

Demographics		
*N*	102	
Gender		
Female	58	56.9%
Male	44	43.1%
Age		
Mean	54.2	18–90
Tobacco exposure		
Current smoker	16	15.7%
Ex-smoker	29	28.4%
Never smoker	57	55.9%
Clinical indication		
Staging for known malignancy	60	58.8%
Lung	18	30%
Colorectal	8	13.3%
Breast	8	13.3%
Skin	6	10%
Multiple myeloma	5	8.3%
Esophageal	4	6.7%
Uterine	2	3.3%
Cervical	2	3.3%
Cancer of unknown origin	2	3.3%
Pituitary	1	1.7%
Brain	1	
Thyroid	1	
Gastric	1	
Ovarian	1	
Investigating potential cancer	10	9.8%
Investigating vasculitis	20	19.6%
Investigating tuberculosis	6	5.8%
Investigating occult infections	6	5.8%

We found a slight preponderance towards female gender (56.9%) with no clinical significance. Age ranged between 18 and 90 years with a median of 55.5. Of the included patients, 16% were current tobacco smokers, while 28% were former smokers. Clinical indication for the scan was as part of staging for other malignancies in 58.8% (*n* = 60), performed for patients with auto-immune diseases such as vasculitis or granulomatous diseases in 19.4% (*n* = 20), suspicion of new diagnosis of cancer in 9.8% (*n* = 10), as investigation for tuberculosis in 5.8% (*n* = 6) and to look occult infection in 5.8% (*n* = 6) of patients. Of the patients who underwent PET/CT for staging of underlying malignancies, 30% (*n* = 18) had lung, 13.3% (*n* = 8) had colorectal, 13.3% (*n* = 8) had breast, 10% (*n* = 6) had melanoma, 8.3% (*n* = 5) had multiple myeloma, 6.7% (*n* = 4) had esophageal, 3.3% (*n* = 2) had uterine and 3.3% (*n* = 2) had cervical cancer. The remaining 11.8% (*n* = 7) of malignancies were cancers of unknown primary, pituitary, brain, thyroid, gastric and ovarian.

Capillary blood glucose levels ranged between 3.1 and 9 mmol/l with an average of 5.5 mmol/l. Patients received on average 363.6 MBq of FDG, and image acquisition time averaged 63 min post-injection.

Oropharyngeal uptake was asymmetrical in 57.8% (*n* = 59). Incidental uptake was more common in the tonsils (55.9%; *n* = 57) than in the base of the tongue (31.4%; *n* = 32), with 12.7% (*n* = 13) of patients demonstrating uptake at both sites (Table 3). SUV_max_ measurements were reported in 51% of scans (*n* = 51) and varied between 3.1 and 25.3 (mean 9.1).

**Table 3:** Results—radiographic findings, follow-up and further investigations (*N* = 102)

Results		
PET uptake		
Asymmetry	59	57.8%
Base of tongue	32	31.4%
Palatine tonsils	57	55.9%
SUX_max_	Median 7.65 (range 3.1–25.3)	
Report recommendations		
Direct visualisation	42	41.2%
Clinical correlation	14	13.7%
Specialist opinion	4	3.9%
Outcomes		
Seen in ENT clinic	25	24.5%
Reassured and discharged	9	36%
Further follow-up arranged	8	32%
Further investigations		
Repeat imaging	15	14.7%
Tissue diagnosis	9	8.8%
Declined further follow-up	1	1%

Official radiology reports described 41.2% (*n* = 42) of findings as likely physiological; however 52.9% (*n* = 54) of reports advised further action. The most frequent recommendation was direct visualisation in 41.2% (*n* = 42), correlation with clinical findings in 13.7% (*n* = 14) and specialty otolaryngology opinion in 3.9% (*n* = 4) of reports. Of these patients, only 24.5% (*n* = 25) were referred and reviewed by an otolaryngologist. Patients reviewed by an ENT specialist were more likely to have asymmetrical uptake (84%) and SUVmax values were on median 7.65 (range 3.1–20.1). Of these, nine patients were reassured and discharged following their first appointment with ENT, eight patients had subsequent follow-up and 15 required an interval PET/CT. The interval scans showed partial or complete resolution in all cases. Furthermore, nine patients proceeded to tissue diagnosis. In the subgroup of patients which underwent biopsy, three had tonsil uptake, four had base of tongue uptake and two had uptake in both regions, uptake was asymmetrical in 66.7, 44% had history of past or current smoking and SUV_max_ values were on median 7.6 (range 3.1–15.3). Biopsies revealed follicular hyperplasia (3), low-grade dysplasia (1), amyloid tissue (1), tongue base cysts (1), reactive lymph node (1), normal lymphoid tissue (1) and metastatic adenocarcinoma (1).

The biopsy positive for metastatic adenocarcinoma belonged to a 73 year-old man with a history of Ivor Lewis esophagectomy 5 years previously, who presented with a new neck lump. The PET/CT showed a soft tissue tumour (SUVmax 4.0) at the right thoracic inlet with extension into the right lobe of the thyroid and trachea, and “faint” activity (SUV_max_ 3.1) in the base of tongue extending to the valeculla, with some soft tissue asymmetry on the non-contrast CT component. Direct visualisation was recommended.

A second patient with a radiologically diagnosed base of tongue tumour was a 66 year-old man with an advanced T4N1 rectal cancer with liver and lung metastases. The PET/CT revealed a left valecullar and tongue base lesion with increased FDG uptake crossing the midline, suggestive of a second primary. Following discussion at the multidisciplinary team meeting, it was decided not to further investigate the new primary in view of the patient’s poor prognosis.

None of the other patients developed oropharyngeal malignancy at a later date. The median follow-up was 28.6 months with a minimum follow-up of 12 months.

## Discussion

Incidental FDG uptake in the head and neck during PET scans is common. However, to the best of our knowledge, no studies have investigated incidental FDG uptake in the oropharynx specifically. Incidental PET uptake in the head and neck was analysed in 16 papers and was shown to occur in 13.8% of scans on average (Table 4) [11–25]. The presence of PET incidentalomas was associated with a significant increase in time to commencement of treatment [14]. Incidental uptake was typically benign, in the form of inflammatory or reactive lymphoid hyperplasia [13–16, 20, 26]. Unexpected foci of uptake on the PET/CT represented malignant deposits from the primary cancer with rates varying between 3 and 50% (average 27%), with rates higher in patients with parotid uptake [24]. Of note, it was shown that SUV_max_ from cervical lymph nodes in patients with malignant incidental lesions was on average higher than in patients with benign findings [23].

**Table 4:** Literature review and study characteristics

First author, year	*n*	Age (years)	Study type, level of evidence	Cohort (underlying pathology)	H&N incidental findings	H&N malignancy	H&N malignancy described
Britt^a^ 2018 [11]	293	62.8	Retrospective level III	H&N cancer	45%	*N* = 1	1 parotid malignancy
Casselden^a^ 2019 [12]	93	34–85	Retrospective level III	H&N cancer	2.1%	None	None
Osman^a^ 2017 [13]	764	68–82	Retrospective level III	Prostate cancer	0.2%	*N* = 1	1 base of tongue SCC
Ali^a^ 2017 [14]	273	38–81	Prospective level III	Lung, oesophageal, H&N, lymphoma, genitourinary, gastric, melanoma, other cancer	NR	*N* = 3	1 parotid adenocarcinoma, 1 laryngeal SCC and 1 hypopharyngeal SCC
Schaarschmid 2017 [15]	81	54.4 ± 15	Retrospective level III	H&N cancer	32%	None	None
Conrad^a^ 2016 [16]	181	26–87	Retrospective level III	Melanoma	1.1%	None	None
Lee, 2016 [17]	317	63	Retrospective level III	Esophageal cancer	NR (nasopharynx only)	*N* = 4	3 hypopharyngeal SCC and 1 oropharyngeal SCC
Cho^a^, 2016 [18]	56,585	58.2 ± 11.4	Retrospective level III	Known cancer (without H&N)	0.7% (piriform sinus only)	*N* = 29	1 metastatic deposit from incidental tonsil SCC
Seo, 2015 [19]	1342	34–78	Retrospective level III	Prostate cancer	2.1% (parotid only)	*N* = 8	8 metastatic deposits from primary malignancy
Williams 2015 [20]	609	NR	Retrospective level III	Lung cancer	12.5%	*N* = 1	1 papillary cancer thyroid
Gobel 2014 [21]	592	22–85	Retrospective level III	Lung cancer	11%	*N* = 4	2 laryngeal SCC, 1 oral cavity SCC; 1 parotid undifferentiated carcinoma and 1 osteosarcoma of the mandible
Patel^a^ 2014 [22]	1846	70	Retrospective level III	Lung cancer	2.6%	*N* = 3	1 tongue SCC
Al-Hakami 2011 [23]	1565	NR	Retrospective level III	Non-H&N cancer	2.4%	*N* = 8	5 thyroid cancer, 2 parotid cancer and 1 cervical node positive for SCC
Heusner 2009 [24]	590	55.4 ± 13.3	Retrospective level III	Non-H&N cancer	60%	*N* = 2	1 palatine tonsil SCC and 1 oral floor SCC
Choi 2005 [25]	547	60.5 ± 10.5	Retrospective level III	New cancer diagnosis	NR	*N* = 7	NR

^a^ Excluded thyroid uptake, *H&N* head and neck, *n* number of patients included in study, *NR* not reported

New, truly positive scans were uncommon. A new malignancy was diagnosed in 0.5–10% of patients with incidental head and neck uptake, more commonly in smokers [17]. Moreover, synchronous cancers were shown to be at a more incipient stage when diagnosed (stages I and II) [17, 19, 21]. Synchronous primaries in the Waldeyer’s ring were rare. There was one case of a tongue base SCC diagnosed following staging investigations with ^68^ Ga prostate specific membrane antigen (PSMA) PET/CT for a prostate cancer [18]. In the series by Cho et al. looking at uptake in the piriform sinus, there was one case of metastatic deposit from an incidental tonsil SCC [23]. Patel et al. diagnosed a further tongue SCC in a patient undergoing FDG PET/CT for a known lung primary, who had frank clinical evidence of a synchronous head and neck malignancy [14]. There was one case of a patient diagnosed with tonsil SCC following evidence of increased but symmetrical uptake (SUV_max_ 3.2) during FDG PET/CT, suggesting that pattern of uptake might not be a useful predictor of potential malignancy [16].

In our unit, the presence of incidental oropharyngeal PET uptake was 0.88%, which is lower compared to the rate of incidental total head and neck uptake of 13.8%. This is indeed a surprizing finding, more so as the incidence of oropharyngeal malignancy is increasing. This perhaps suggests increasing expertise with PET imaging leading to a reduced number of reporting of incidental lesions which reveals the subjectivity in interpreting these scans. This might be a reflection of recent improvements in PET/CT scanners with innovations in detection technology and spatial resolution [27]. There has been a drastic change from analogue to digital equipment which can be seen in the adoption of digital detectors. These have been shown to reduce noise and improve lesion detectability and diagnostic confidence and may reduce the number of false positives [28].

The rate of incidental oropharyngeal PET uptake which harboured malignancy in this study was 1.9% which is slightly lower than the rates found in the head and neck literature review. This is certainly much lower than the rates documented in the thyroid [29], parotid [24] and piriform sinus [23]. This high false positive rate can be party explained by the intrinsic nature of immune tissue which can be often found in a reactive phase. These findings are somewhat reassuring, suggesting that the majority of incidental PET uptake is benign and only a very small number of patients will present with true malignancy. Furthermore, the rate of positive malignancy in patients undergoing tissue biopsy was low at 11% suggestive that the rate of false positives on PET/CT is still high. Therefore, there might be a case for acquiring magnetic resonance imaging to further characterise these lesions prior to tissue diagnosis. Nevertheless, as oropharyngeal malignancy becomes more common, one cannot be too careful in further investigation of these patients.

Whereas guidelines exist for managing incidental FDG uptake in the parotid, nasopharynx, esophagus, pancreas, uterus and ovaries, no consensus yet exists for managing oropharyngeal uptake [30]. With regards to uptake in the palatine tonsils, this was shown to be on mean SUV_max_ higher in SCC (9.36 + or −4.54) than in contralateral healthy tonsils (2.54 + or −0.88; *p* < 0.0001) or in control subjects (2.98 + or −1.08; *p* < 0.0001) [31]. A further study proposes that a difference of above SUV_max_ 1.6 between the two tonsils suggests a site of biopsy in SCC of unknown primary [32]. These guidelines, however, are limited by small numbers and the poor validity of SUV_max_ values across different centres and therefore results lose comparability. Despite this, the current findings suggest that the risk of malignancy is small and clinicians can find reassurance. In addition, many of the patients undergoing PET imaging will undergo subsequent follow-up as part of their original pathology which presents an opportunity for monitoring of changes.

The cost implications involved in further investigating these radiological findings also needs discussed. Adams et al. calculated costs of follow-up in 215 consecutive patients undergoing PET-CT in Ontario, Canada [33]. Of these, 161 (74.9%) had incidental findings and 75 reports had recommendations for further follow-up. The cost for the recommended investigations was calculated at $127.56 (US Dollars) per each patient undergoing PET–CT. However, this did not take into account any costs associated with new referrals and appointments. The cost of new appointments in the Ears, Nose and Throat department in Imperial College Healthcare NHS Trust, UK average at £120 (GBP) excluding endoscopic examination, suggesting that costs might be as high as £220 for patients requiring follow-up of incidental lesions within our unit.

## Limitations

This is the first study investigating incidental PET/CT uptake in the oropharynx and we acknowledge certain limitations. Despite the considerable time-frame, the number of included patients remains small. Organising large multi-centre trials, however, remains difficult in view of the heterogeneity of PET scanners and high variability in local radiology expertise. Furthermore, SUV values vary depending on image quality (noise, resolution), tumour size (with smaller tumours demonstrating lower SUV), as well as on the acquisition and processing of images. As SUV vary among different institutions and studies, results are often non-comparable [34, 35].

Due to the wide time-frame of the present study, there was also variation in the available PET technology, scan report and recommendation and examination, investigation and follow-up of included patients.

## Conclusion

Oropharyngeal SCC is becoming increasingly common and early diagnosis is of paramount importance [36]. However, the current study suggests that, whereas incidental FDG uptake in the Waldeyer’s ring is common on PET imaging, it is unlikely to harbour any malignancy. When oropharyngeal malignancy occurs, it is typically associated with clinical signs and structural changes on the CT component. This emphasises the importance of direct visualisation when investigating incidental oropharyngeal PET uptake. The development of a risk-stratification model for patients with unexplained oropharyngeal uptake who require a second opinion needs to be addressed. High risk factors include tobacco consumption and alcohol intake, upper gastrointestinal tract malignancies [37] and lung malignancies [38, 39]. Despite its variability, SUV_max_ has been shown to be high in cases of true malignancy in the palatine and lingual tonsils and, thus, should be taken into consideration when assessing risk. Furthermore, radiology reports often comment on suspicious uptake despite apparent symmetry and give recommendations on further follow-up or repeat imaging. This can be an invaluable tool in stratifying high-risk patients and reducing unnecessary investigations. Lastly, with the advent of liquid biopsies in diagnosing malignancy and detection of HPV infection, clinicians can further predict head and neck malignancy risk [40]. However, developing a feasible and accurate risk-stratification model for patients with incidental uptake in the oropharynx requires additional work.

## Data Availability

Anonymised data available upon request.

## References

[1] Head and neck cancers incidence statistics | Cancer Research UK. Cancer Research UK, https://www.cancerresearchuk.org/health-professional/cancer-statistics/statistics-by-cancer-type/head-and-neck-cancers/incidence#heading-Four 2016, Accessed on 24 March 2019

[2] Hashibe M, Brennan P, Chuang S, Boccia (2009). Interaction between tobacco and alcohol use and the risk of head and neck cancer pooled analysis in the international head and neck cancer epidemiology consortium. Cancer Epidemiol Biomarkers Prev.

[3] Waridel F, Estreicher A, Bron L (1997). Field cancerisation and polyclonal p53 mutation in the upper aero- digestive tract. Oncogene.

[4] Morris LGT, Sikora AG, Hayes RB (2011). Anatomic sites at elevated risk of second primary cancer after an index head and neck cancer. Cancer Causes Control.

[5] Bugter O, van de Ven SEM, Hardillo JA (2019). Early detection of esophageal second primary tumors using Lugol chromoendoscopy in patients with head and neck cancer: a systematic review and meta-analysis. Head Neck.

[6] Dirix P, Vandecaveye V, De Keyzer F (2009). Dose painting in radiotherapy for head and neck squamous cell carcinoma: value of repeated functional imaging with (18)F-FDG PET, (18)F-fluoromisonidazole PET, diffusion-weighted MRI, and dynamic contrast-enhanced MRI. J Nucl Med.

[7] Nakamura S, Okochi K, Murata Y (2009). [18F]Fluorodeoxyglucose-PET/CT differentiation between physiological and pathological accumulations in head and neck. Nucl Med Commun.

[8] Purohit BS, Ailianou A, Dulguerov N (2014). FDG-PET/CT pitfalls in oncological head and neck imaging. Insights Imaging.

[9] Liu Y, Ghesani NV, Zuckier LS (2010). Physiology and pathophysiology of incidental findings detected on FDG-PET scintigraphy. Semin Nucl Med.

[10] Nakamoto Y, Tatsumi M, Hammoud D (2005). Normal FDG distribution patterns in the head and neck: PET/CT evaluation. Radiology.

[11] Britt CJ, Maas AM, Kennedy TA (2018). Incidental findings on FDG PET/CT in head and neck cancer. Otolaryngol Neck Surg.

[12] Casselden E, Sheerin F, Winter SC (2019). Incidental findings on 18 FDG PET CT in head and neck cancer. A retrospective case control study of incidental findings on 18 FDG PET CT in patients with head and neck cancer. Eur Arch Oto Rhino Laryngol.

[13] Gobel Y, Valette G, Abgral R (2014). Interpretation of suspect head and neck fixations seen on PET/CT in lung cancer. Eur Ann Otorhinolaryngol Head Neck Dis.

[14] Patel A, Perry T, Hunt I (2014). Should we routinely investigate incidental head and neck findings on 18-fluorodeoxyglucose positron emission tomography in patients being staged for non-small cell lung cancer?. A Retro Anal Thorac Cardiovasc Surg.

[15] Al-Hakami HA, Makis W, Anand S (2011). Head and neck incidentalomas on positron emission tomographic scanning: ignore or investigate?. J Otolaryngol Head Neck Surg.

[16] Heusner TA, Hahn S, Hamami ME (2009). Incidental head and neck 18F-FDG uptake on PET/CT without corresponding morphological lesion: early predictor of cancer development?. Eur J Nucl Med Mol Imaging.

[17] Choi JY, Lee KS, Kwon OJ (2005). Improved detection of second primary cancer using integrated [18 F] fluorodeoxyglucose positron emission tomography and computed tomography for initial tumor staging. J Clin Oncol.

[18] Osman MM, Iravani A, Hicks RJ (2017). Detection of synchronous primary malignancies with 68Ga-labeled prostate-specific membrane antigen PET/CT in patients with prostate cancer: frequency in 764 patients. J Nucl Med.

[19] Ali SA, Hamed MAE (2017). The diagnostic efficacy of whole body 18F-FDG PET CT in detection of unexpected second primary malignancy in cancer patients. Egypt J Radiol Nucl Med.

[20] Schaarschmidt BM, Gomez B, Buchbender C (2017). Is integrated 18F-FDG PET/MRI superior to 18F-FDG PET/CT in the differentiation of incidental tracer uptake in the head and neck area?. Diagn Interv Radiol.

[21] Conrad F, Winkens T, Kaatz M (2016). Retrospective chart analysis of incidental findings detected by (18) F-fluorodeoxyglucose-PET/CT in patients with cutaneous malignant melanoma. J Dtsch Dermatol Ges.

[22] Lee JS, Ahn JY, Choi KD, et al. Synchronous second primary cancers in patients with squamous esophageal cancer: clinical features and survival outcome. Korean J Intern Med; 31, http://www.ncbi.nlm.nih.gov/pubmed/26864297 2016, Accessed on 9 December 2018 10.3904/kjim.2014.182 PMC4773710 26864297

[23] Cho YS, Moon SH, Choi JY (2016). Clinical significance of incidental 18F-FDG uptake in the pyriform sinus detected by PET/CT. Clin Nucl Med.

[24] Seo YL, Yoon DY, Baek S (2015). Incidental focal FDG uptake in the parotid glands on PET/CT in patients with head and neck malignancy. Eur Radiol.

[25] Williams SP, Kinshuck AJ, Williams C (2015). Incidental head and neck findings on 18F-fluoro-deoxy-glucose positron emission tomography computed tomography. J Laryngol Otol.

[26] Williams SP, Kinshuck AJ, Williams C (2015). Incidental head and neck findings on ^18^ F-fluoro-deoxy-glucose positron emission tomography computed tomography. J Laryngol Otol.

[27] Vandenberghe S, Moskal P, Karp JS (2020). 2020 State of the art in total body PET. EJNMMI Phys.

[28] Schillaci O, Urbano N (2019). Digital PET/CT: a new intriguing chance for clinical nuclear medicine and personalized molecular imaging. Eur J Nucl Med Mol Imaging.

[29] Agrawal K, Weaver J, Ul-Hassan F (2015). Incidence and significance of incidental focal thyroid uptake on ^18^F-FDG PET study in a large patient cohort: retrospective single-centre experience in the United Kingdom. Eur Thyroid J.

[30] Pencharz D, Nathan M, Wagner TL (2017). Evidence based management of incidental focal uptake of fluorodeoxyglucose on PET-CT. Br J Radiol.

[31] Davison JM, Ozonoff A, Imsande HM (2010). Squamous cell carcinoma of the palatine tonsils: FDG standardized uptake value ratio as a biomarker to differentiate tonsillar carcinoma from physiologic uptake. Radiology.

[32] Pencharz D, Dunn J, Connor S (2019). Palatine tonsil SUVmax on FDG PET-CT as a discriminator between benign and malignant tonsils in patients with and without head and neck squamous cell carcinoma of unknown primary. Clin Radiol.

[33] Adams SJ, Rakheja R, Bryce R (2018). Incidence and economic impact of incidental findings on 18F-FDG PET/CT imaging. Can Assoc Radiol J.

[34] Kinahan PE, Fletcher JW (2010). Positron emission tomography-computed tomography standardized uptake values in clinical practice and assessing response to therapy. Semin Ultrasound CT MR.

[35] Boellaard R, Krak NC, Hoekstra OS (2004). Effects of noise, image resolution, and ROI definition on the accuracy of standard uptake values: a simulation study. J Nucl Med.

[36] Anderson CM, Chang T, Graham MM (2015). Change of maximum standardized uptake value slope in dynamic triphasic [18F]-fluorodeoxyglucose positron emission tomography/computed tomography distinguishes malignancy from postradiation inflammation in head-and-neck squamous cell carcinoma: a prospective trial. Int J Radiat Oncol.

[37] Muto M, Hironaka S, Nakane M (2002). Association of multiple Lugol-voiding lesions with synchronous and metachronous esophageal squamous cell carcinoma in patients with head and neck cancer. Gastrointest Endosc.

[38] Wax MK, Myers LL, Gabalski EC (2002). Positron emission tomography in the evaluation of synchronous lung lesions in patients with untreated head and neck cancer. Arch Otolaryngol Head Neck Surg.

[39] Schwartz LH, Ozsahin M, Zhang GN (1994). Synchronous and metachronous head and neck carcinomas. Cancer.

[40] Qureishi A, Mawby T, Fraser L (2017). Current and future techniques for human papilloma virus (HPV) testing in oropharyngeal squamous cell carcinoma. Eur Arch Otorhinolaryngol.

